# *Ixodes holocyclus* Tick-Transmitted Human Pathogens in North-Eastern New South Wales, Australia

**DOI:** 10.3390/tropicalmed1010004

**Published:** 2016-08-11

**Authors:** Stephen R. Graves, Chrissie Jackson, Hazizul Hussain-Yusuf, Gemma Vincent, Chelsea Nguyen, John Stenos, Maurice Webster

**Affiliations:** 1 Australian Rickettsial Reference Laboratory, Geelong, 3220 VIC, Australia; HAZIZUL@BarwonHealth.org.au (H.H.-Y.); gvince@barwonhealth.org.au (G.V.); CHELSEAN@BarwonHealth.org.au (C.N.); johns@barwonhealth.org.au (J.S.); 2 Wongaburra Research Centre, Vet^x^ Research, Casino, NSW 2470, Australia; cjackson@vetx.com.au (C.J.); mwebster@vetx.com.au (M.W.)

**Keywords:** tick, *Ixodes holocyclus*, pathogens, Australia

## Abstract

A group of 14 persons who live in an area of Australia endemic for the Australian paralysis tick, *Ixodes holocyclus*, and who were involved in regularly collecting and handling these ticks, was examined for antibodies to tick-transmitted bacterial pathogens. Five (36%) had antibodies to *Coxiella burnetii*, the causative agent of Q fever and three (21%) had antibodies to spotted fever group (SFG) rickettsiae (*Rickettsia* spp). None had antibodies to *Ehrlichia*, *Anaplasma*, *Orientia*, or *Borrelia* (Lymedisease) suggesting that they had not been exposed to these bacteria. A total of 149 *I. holocyclus* ticks were examined for the citrate synthase (*gltA*) gene of the SFG rickettsiae and the *com1* gene of *C. burnetii*; 23 (15.4%) ticks were positive for *Rickettsia* spp. and 8 (5.6%) positive for *Coxiella* spp. Sequencing of fragments of the *gltA* gene and the 17 kDa antigen gene from a selection of the ticks showed 99% and 100% homology, respectively, to *Rickettsia australis*, the bacterium causing Queenslandtick typhus. Thus, it appears that persons bitten by *I. holocyclus* in NE NSW, Australia have an approximate one in six risk of being infected with *R. australis*. Risks of Q fever were also high in this region but this may have been due to exposure by aerosol from the environment rather than by tick bite. A subset of 74 *I. holocyclus* ticks were further examined for DNA from *Borrelia* spp., *Anaplasma* spp. and *Ehrlichia* spp. but none was positive. Some of these recognised human bacterial pathogens associated with ticks may not be present in this Australian tick species from northeastern New South Wales.

## 1. Introduction

The number and diversity of human arthropod-borne infections following tick bites are second only to those following mosquito bites [1]. In Australia, a number of tick-borne diseases are endemic. These include Q fever caused by *Coxiella burnetii*, Queensland tick typhus caused by *Rickettsia australis*, Flinders Island spotted fever caused by *R. honei*, and Australian spotted fever caused by *R. honei subsp marmionii* [2,3,4]. The paralysis tick *Ixodes holocyclus* from central coast New South Wales has been shown to contain a number of potential human pathogens [5], but the risk of infection to persons who are bitten by this tick is not known. This is considered worthy of investigation particularly as this tick commonly bites humans in eastern Australia.

In this study staff from a companion animal contract research organisation (Wongaburra Research Centre, operated by Vet^X^ Research), located at Casino, in northeastern New South Wales and a group of associated tick collectors, were examined for antibodies to known tick-transmitted bacterial infections. In addition, individual *I. holocyclus* ticks were examined for the presence of bacterial pathogens by qPCR amplification of unique bacterial genes.

## 2. Materials and Methods

Persons who worked at, or were tick collectors for, Wongaburra Research Centre took part in this project as they had considerable exposure to *I. holocyclus* during the course of their work collecting and applying these ticks to dogs.

This study was discussed with the local Human Ethics Committee but a formal review was not required as all 14 persons had actively sought to be involved for their own interest. Nevertheless, signed informed consent was obtained from all participants.

Fourteen persons had their serum tested for antibodies to *Rickettsia* spp., *C. burnetii*, *Orientia tsutsugamushi*, *Anaplasma* spp., *Ehrlichia* spp., and *Borrelia burgdorferi* according to the standard serological diagnostic assays in use at the Australian Rickettsial Reference Laboratory (ARRL) in Geelong, Victoria, Australia. These assays were mainly immunofluorescence. [6] For antibodies to *C. burnetii* a titre of ≥50 was considered positive, for all three immunoglobulin classes (IgM, IgG, and IgA) and against both antigenic phases (1 & 2) of *C. burnetii*. For antibodies to *Rickettsia* spp. a titre of 128 was considered to be positive. This assay does not differentiate between recent and past infection, but indicates exposure to the rickettsial pathogen. *I. holocyclus* ticks were collected from the local environment around Casino and used as part of the Wongaburra in-house research program. After use, ticks were sent to the ARRL where DNA was extracted and qPCR assays were performed to detect the citrate synthase (*gltA*) gene from *Rickettsia* spp. and the *com1* gene from *C. burnetii*. Fragments of the citrate synthase and 17 kDa antigen gene from a selection of *Rickettsia* spp. positive tick samples were amplified as previously described [7,8] and sequenced. A BLAST analysis was performed on the resulting sequences to determine the rickettsial species. Analysis of a subset of 74 ticks was undertaken by qPCR targeting DNA from *Borrelia* spp. [9]. Universal primers that detected both *Anaplasma* spp. and *Ehrlichia* spp. [10] were used to look for these bacteria.

## 3. Results

Of the 14 participants in the study, five (36%) had antibodies to *C. burnetii* and three (21%) had antibodies to *Rickettsia* spp. (Table 1). For the five persons with antibodies to *C. burnetii* all had serological patterns consistent with past exposure (Table 2). The three persons with rickettsial antibodies, all had titres of 128 or 256, to spotted fever group rickettsiae, indicating likely past exposure (Table 3). One participant (#12) also had antibodies to typhus group (TG) rickettsiae, which may have indicated past exposure or else it was a serological cross-reaction to TG after SFG rickettsiae exposure. Australian TG rickettsiae are not known to be transmitted by ticks. No participant had antibodies to *O. tsutsugamushi*, the causative agent of scrub typhus. No participant had antibodies to *Borrelia burgdorferi*, *Anaplasma phagocytophilum*, or *Ehrlichia chaffeensis*, (Table 1).

Of the 149 individual *I. holocyclus* ticks tested by qPCR, 23 (15.4%) contained the *gltA* gene characteristic of SFG/TG rickettsiae. From three of the *gltA*-positive ticks a larger fragment of the gene was amplified and sequenced and shown in each case to have 99% homology with *R. australis*. One of these ticks had another rickettsial-specific gene (17 kDa) detected, amplified, and sequenced. It was shown to be 100% homologous to *R. australis*. DNA from 143 of the 149 ticks was also tested by qPCR for the presence of the *com1* gene of *C. burnetii* and 8 (5.6%) ticks were positive. DNA from 74 of the 149 ticks was also tested for genes of *Borrelia* spp., *Anaplasma* spp., and *Ehrlichia* spp., but all ticks were negative.

## 4. Discussion

Patients often report being unwell after receiving a tick bite in Australia, with a range of symptoms reported, some of which are consistent with infection. However, there is no clear understanding of the infectious risk following tick bite in Australia, neither with respect to different tick species, several of which are known to bite humans, nor the geographic location within Australia, nor the microbial cause of the infectious disease. While rickettsial infection is well recognised in Australia, other tick-borne infections, such as ehrlichiosis, human anaplasmosis, and human borreliosis, including Lyme disease, have not been convincingly detected and may, in fact, be absent from Australia. A recent study [5] of 196 *I. holocyclus* ticks from central coast New South Wales detected several potential human pathogens, including relapsing fever *Borrelia*, *Bartonella*, *Clostridium*, *Rickettsia*, *Leptospira* and “Candidatus *Neoehrlichia*”. However, the significance for human health of these findings is not yet clear, as the detection of a microbe in a tick does not imply that it can be transmitted to a human, nor that it can cause illness in a person.

Several Australian tick species are known to bite humans (*I. holocycylus*, *I. tasmani*, *I. cornuatus*, *Amblyomma triguttatum*, and *Bothriocroton hydrosauri*), but the paralysis tick, *I. holocyclus*, is probably the main culprit. This is partly because it is endemic to the eastern seaboard of Australia from Cooktown in Queensland, to Victoria in the south [11,12,13], which is where the human population of Australia is most heavily concentrated. Tick-bite in the well-vegetated gardens and bushland of the northern suburbs of Sydney is a well-recognised problem [14,15], and local doctors in that region of Australia are often called upon to deal with the clinical sequelae, be it anaphylaxis (very rare), infection (rare), or dermatological conditions and/or anxiety (very common). The diagnosis of post-tick bite Lyme disease is sometimes made on clinical grounds alone, without proper laboratory support for that particular diagnosis.

The staff and tick collectors of the Wongaburra Research Centre work with *I. holocyclus* as part of their routine duties. One may assume that they would be bitten by this tick from time to time, probably more often than the average person living in Casino. Hence, they were a cohort worth investigating for tick-transmitted infections, due to their presumed higher risk of exposure. As they were all currently healthy there was no purpose in testing them for the presence of tick-transmitted microbes or microbial DNA. However, testing for the presence of antibodies was considered to be a relatively simple way of detecting past microbial exposure. It is a crude measure, as antibody levels decay with time, so a negative antibody result does not reliably rule out prior exposure. However, a positive result, provided the serological assay has adequate specificity, is a good indicator of prior exposure to that microbe.

That 5/14 (36%) of persons were positive for *C. burnetii* antibodies was not surprising as this region of Australia is endemic for Q fever [16]. Other similar rural regions of Australia [17,18,19] also have a high incidence of Q fever and high seroprevalence to this bacterium. It is a zoonotic pathogen transmitted from infected vertebrate animals (both domestic and native [20,21]) via contaminated aerosols. Nevertheless, tick bite cannot be ruled out and a previous report has suggested Q fever may have been transmitted this way [22].

The situation with respect to rickettsial infection is a little clearer, as tick-bite, usually from *I. holocyclus*, is the only recognised mode of human infection by *R. australis* [23,24,25,26,27,28,29,30,31,32,33]. As 15.4% of these ticks examined in this study contained rickettsial DNA and 21% of persons in the study had antibodies to *Rickettsia* spp., it seems reasonable to postulate that there is approximately a one in six risk of rickettsial infection following the bite of an *I. holocyclus* tick in this part of Australia. Of course, this assumes that the tick goes undetected and remains attached to the patient for the many hours it takes before transmission of *R. australis* occurs from the tick to the person. A recent study [34] of the two main species of ticks biting humans in Australia (*I. holocyclus* on the east coast of Australia and *Ambylomma triguttatum* in the SW of Western Australia) demonstrated that 30.5% of the former contained DNA from the genus *Rickettsia*.

A small study such as this, in a localised region of Australia and examining only one species of human-biting tick, cannot rule out other tick-transmitted bacterial infections in other parts of Australia or involving other species of tick [35]. For example, in Western Australia, the epidemiology of tick-transmitted human infections may be quite different to the eastern seaboard. However, in NE NSW, the tick *I. holocyclus* appears not to harbour, nor transmit to humans, bacterial pathogens of the genera *Borrelia*, *Ehrlichia*, and *Anaplasma*. Further studies, in other parts of Australia are warranted to investigate this further. The absence of antibodies to *O. tsutsugamushi* in the 14 participants was to be expected as scrub typhus is a tropical rickettsial infection transmitted by a larval form of mite, not a tick, and is not known to occur in NSW.

Clinicians are often asked whether antibiotics should be given prophylactically to patients who have been bitten by a tick but who are currently quite well [36]. Until now the answer has been based on guesswork only, as the prevalence of pathogens in this tick species has been unknown. Based on the data from this current study and particularly if the tick has been removed from the patient within a few hours of attachment (although this is often difficult to ascertain), the use of prophylactic antibiotics to prevent the development of tick-transmitted bacterial infection in the patient is not recommended. Locally applied disinfectant to the bite site after proper removal of the tick and advice to return for further medical care if a fever should develop, is probably the most appropriate medical advice to the patient.

## 5. Conclusions

Persons bitten by the paralysis tick *I. holocyclus* in Northeastern NSW, Australia, are at risk of infection with *R. australis*, causing Queensland tick typhus and, to a lesser extent, *C. burnetii*, causing Q fever.

The risk of rickettsial infection following the bite of the tick *I. holocyclus* in NE NSW appears to be about one in six.

There was no evidence of DNA from *Anaplasma* spp. or *Ehrlichia* spp. in the ticks examined.

There was also no evidence of Lyme disease bacterial DNA in the *I. holocyclus* ticks examined nor of antibodies to Lyme disease bacteria (*Borrelia* spp.) in the participants, all of whom had significant exposure to the tick *I. holocyclus*.

## Figures and Tables

**Table 1:** Serology for tick-transmitted bacteria of 14 persons living in a region of NSW endemic for the tick *Ixodes holocyclus*.

Participant Number	SEX	AGE	SEROLOGY (antibodies to)					
			*Coxiella burnetii* (Q fever)	*Rickettsia* spp. (rickettsiosis)	*Orientia tsutsugamushi* (scrub typhus)	*Anaplasma phagocytophilum* (anaplasmosis)	*Ehrlichia chaffeensis* (ehrlichiosis)	*Borrelia burgdorferi* (Lyme disease)
1	F	49	−	−	−	−	−	−
2	F	50	−	+	−	−	−	−
3	F	47	+	−	−	−	−	−
4	F	66	−	−	−	−	−	−
5	M	48	+	−	−	−	−	−
6	F	32	+	−	−	−	−	−
7	F	24	−	−	−	−	−	−
8	F	32	−	−	−	−	−	−
9	M	65	−	−	−	−	−	−
10	F	18	−	−	−	−	−	−
11	M	51	+	−	−	−	−	−
12	F	29	−	+	−	−	−	−
13	F	54	+	−	−	−	−	−
14	M	61	−	+	−	−	−	−
			5/14 (36%)	3/14 (21%)	0/14 (0%)	0/14 (0%)	0/14 (0%)	0/14 (0%)

F: female; M: male; +: detected; −: not detected.

**Table 2:** Positive Q fever serology in five persons living in a region of NSW endemic for the tick *Ixodes holocyclus*.

Participant Number	Antibody Titres to *Coxiella burnetii*					
	Phase 2 Antigen			Phase 1 Antigen		
	IgM	IgG	IgA	IgM	IgG	IgA
3	−	800	50	50	200	−
5	100	800	−	−	50	−
6	−	50	−	−	−	−
11	−	1600	200	−	−	100
13	−	200	−	−	−	−
Positives	1/5	5/5	2/5	1/5	2/5	1/5

−: not detected.

**Table 3:** Positive rickettsial serology in three persons living in a region of NSW endemic for the tick *Ixodes holocyclus*.

Participant Number	Antibody Titres to Rickettsial Group		
	Spotted Fever Group (e.g., *Rickettsia australis*)	Typhus Group (e.g., *Rickettsia typhi*)	Scrub Typhus Group (e.g., *Orientia tsutsugamushi*)
2	128	−	−
12	128	128	−
14	256	−	−
Positives	3/3	1/3	0/3

−: not detected.
